# Cyber-ethnography of cannabis marketing on social media

**DOI:** 10.1186/s13011-021-00359-w

**Published:** 2021-04-26

**Authors:** Marina C. Jenkins, Lauren Kelly, Kole Binger, Megan A. Moreno

**Affiliations:** Department of Pediatrics, University of Wisconsin-Madison, 2870 University Ave, Suite 200, Madison, WI 53705 USA

**Keywords:** Social media, Ethnography, Adolescent health, Cannabis, Marketing

## Abstract

**Background:**

Since 2012, several states have legalized non-medical cannabis, and cannabis businesses have used social media as a primary form of marketing. There are concerns that social media cannabis exposure may reach underage viewers. Our objective was to identify how cannabis businesses cultivate an online presence and exert influence that may reach youth.

**Methods:**

We chose a cyber-ethnographic approach to explore cannabis retailers on social media. We searched cannabis retailers with Facebook and Instagram presence from Alaska, Oregon, Colorado, and Washington, and identified 28 social media business profiles. One year of content was evaluated from each profile. In-depth, observational field notes were collected from researchers immersed in data collection on business profiles. Field notes were analyzed to uncover common themes associated with social media cannabis marketing.

**Results:**

A total of 14 businesses were evaluated across both Facebook and Instagram, resulting in 14 sets of combined field notes. A major theme was Normalization of Cannabis, involving both Broad Appeal and Specific Targeting.

**Conclusions:**

It is concerning that Normalization of Cannabis by cannabis businesses may increase cannabis acceptability among youth. In a digital world where the majority of youth are spending time online, it is important for policymakers to examine additional restrictions for cannabis businesses marketing through social media.

**Supplementary Information:**

The online version contains supplementary material available at 10.1186/s13011-021-00359-w.

## Background

In November 2012, Washington State passed Initiative No. 502 (I-502) which legalized non-medical, adult cannabis use. Since then, 11 states and Washington D.C. have approved the use of cannabis for non-medical use, and the topic of legalization continues to generate new policy measures [1–5]. Legalization in specific states along with increased acceptance of cannabis use among adults has generated public concern regarding youth and exposure to cannabis [6]. U.S. adolescents report using cannabis at a rate of 15% for past-30 days and 30% lifetime use [7, 8]. Adolescents may be at increased risk for potential negative consequences related to cannabis use including academic and work difficulties, mental health concerns, progression to other drugs, and Cannabis Use Disorder [9–13]. More recently, cannabis businesses have begun to promote their products on social media [14].

### Social media marketing and youth exposure

Social media use is popular among adolescents, the majority of youth report being online “almost constantly” [5]. Social media platforms such as Facebook and Instagram prohibit paid advertisements for cannabis; however, cannabis businesses can create promotional profiles or “business profiles” where they are able to interact with customers and promote their products. Customers can engage with cannabis social media profiles in a variety of ways, including liking, sharing, or commenting on posts and/or becoming a “follower,” thus becoming regularly exposed to cannabis content. Legislation in some states, but not all, prevent cannabis businesses from engaging in social media marketing unless less than one third of the audience is under the age of 21 [3, 4]. For example, Oregon, Washington and Colorado have these audience restrictions, while Alaska does not. Even for states with restrictions, there is evidence that age restrictions for online content may be ineffective in preventing youth exposure to alcohol and tobacco content [15–17]. This issue may apply to online cannabis content as well. Therefore, it is important to explore social media content from cannabis businesses as youth may be at risk of exposure.

### Substance marketing

Previous studies of tobacco and alcohol have shown that adolescents are susceptible to influences from media and marketing. Adolescents have been shown to be at increased risk for problematic substance use after exposure to advertisements [18–21]. Tobacco and alcohol companies utilize a variety of marketing techniques that may appeal to youth, including the use of lifestyle appeal, recreational activities, and targeting specific audiences [22–24]. The American Academy of Pediatrics proposed that up to one third of adolescent tobacco and alcohol use may be attributed to marketing [25]. If cannabis retailers also utilize techniques such as targeting, these findings suggest that marketing for cannabis may have a similar concerning impact on use among youth. Research has demonstrated that the majority of youth (90%) report exposure to some form of cannabis marketing. Furthermore, exposure was associated with increased odds of past-year cannabis use [26]. A previous study of cannabis marketing found a higher frequency of passive exposure to cannabis ads, or exposure by those not actively seeking cannabis ads, (66%) compared to actively seeking ads (31%) [27]. Prevention strategies for cannabis promotions on social media suggested by youth included more restrictions around social media content for youth under the age of 21 [28]. Previous studies using content analysis have illustrated youth and social media user perceptions of cannabis marketing practices across websites and social media platforms, including YouTube and Twitter. Specifically, study findings revealed a positive perception of cannabis among YouTube and Twitter users along with normalization of cannabis use and easy accessibility by youth [29, 30]. While these studies identify the types of advertisements and potential risks of youth exposure to online cannabis marketing, additional research is needed to better understand the content shared by cannabis businesses specifically on social media.

Few studies have examined cannabis marketing strategies on social media using qualitative methods. One study used content analysis to better understand cannabis marketing content on social media by examining adherence to cannabis advertising regulations on social media in Washington State [31]. This study relied on discrete, predetermined categories fitting for policy application, however thematic elements of posts that may be influential to youth were not captured using this method. For example, this content analysis study captured categories such as inclusion of required warnings based on policy language, but could not capture emergent categories or themes such as tone. Thus, a new strategy is needed for evaluating more complex or emergent themes associated with online cannabis marketing content. The present study expands upon these previous findings to examine marketing practices of cannabis retailers on Facebook and Instagram directly rather than examining viewer perceptions. These specific platforms are regularly used by cannabis businesses for marketing purposes [29] and were highly popular with youth at the time of this study [5].

### Cyber-ethnography

With the popularity of social media [5] and concerns over youth exposure to cannabis marketing, innovative methods are needed for understanding cannabis marketing content on social media. One innovative approach involves the use of ethnography, a research methodology for understanding the patterns, habits and experiences of a culture-sharing group [32]. In an ethnography, participant observation may involve the researcher as completely detached from participants (complete observer) to fully immersed in the culture they are observing (complete participant) [32]. Sub-types of ethnography have been created to adapt the methodology to apply to digital technologies [33, 34]. One such method of ethnography is cyber-ethnography, or ethnography of technologically facilitated interactions, where individuals are considered to be co-located in an online space [35]. Using social media as the research site, cyber-ethnography recognizes the fluidity of ethnographic places and the uniqueness of online interactions, and can be used to understand cannabis business brand strategies in online marketing [36].

Previous research using ethnography to study adolescent substance use has focused primarily on understanding adolescent attitudes and behaviors towards drug use [36–39].. Cyber-ethnography remains rare as an approach for studying substance use. One previous study examined alcohol consumption [40], and no studies have examined drug use. Since cannabis use is illegal for adolescents, ethical implications for research require a unique approach to studying adolescent substance use, one that may involve a more detached approach. A previous study using cyber-ethnography found that youth used social networking sites to create social identities and digital spaces toward normalizing alcohol consumption [40]. This study is an example of complete observer cyber-ethnography, where researchers did not interact with participants and ethnography was conducted solely online through analysis of participant social media posts.

For our study, cyber-ethnography was selected as an appropriate method to capture complex or emergent themes associated with online cannabis marketing content. The purpose of this study was to use cyber-ethnography to evaluate how cannabis businesses cultivate an online presence and exert influence that may reach youth.

## Methods

### Study design

We applied multi-sitecyber-ethnography to social media profiles maintained by retail cannabis businesses. This study was determined to be exempt by the University of Wisconsin Institutional Review Board as it involved evaluation of publicly available information.

### Profile identification

We identified retail cannabis businesses with Facebook and Instagram presence from Alaska, Oregon, Colorado, and Washington, in order to have a representative sample of states where non-medical cannabis is legalized. We prioritized states in which legalization had been present for a longer period of time, such that enough time had elapsed for states to open retail businesses and those businesses to establish social media profiles with at least a year of content.

Data for this study were obtained from two social media sites, Facebook and Instagram, which are among the most popular social media sites for businesses using social media, as well as youth at the time of this study [41]. Based on pilot testing with various keyword options, potential retail cannabis businesses were identified through a Facebook search using the terms [retail cannabis + state name] under Pages and Local Business or Place. The “About” section or business name from each profile was evaluated to confirm that business’s focus on retail, non-medical cannabis sales. We then determined whether the business had profiles on both Facebook and Instagram. If so, we evaluated further inclusion criteria: business location in the appropriate state, content in English, posting twice within the past two months to ensure the profile was still active, and having maintained both social media profiles since June 2017 so that a full year of content was available for evaluation. Of the businesses that met inclusion criteria, the top 5 most popular profiles were selected for each state based on total followers on Facebook and Instagram. Sixteen businesses initially met search criteria; however, two businesses had profiles removed before data collection began and were removed from our sample. The final sample included 28 social media profiles from 14 businesses. One year of content, from June 2017 through May 2018, was evaluated from each profile.

### Role of investigator

In this study, investigators acted as complete observers, where the observer remains detached from the participants who are unaware they are being observed [42]. This allows for an unbiased snapshot of typical practices of those being observed. We chose this type of participant observation because we were interested in acting as observers of the retail profiles, similar to how we hypothesized that adolescents may experience online cannabis marketing. Carspecken’s five stages of qualitative research was used as a framework for our study procedures [43].

### Data collection

#### Stage 1 – compiling the primary record

This step is comprised of fieldwork by observers and recording of field notes [43]. Ideally, ethnography should begin with a conscious attitude of almost complete ignorance, and the researcher then builds a “thick description” by unobtrusively observing social practices. The goal is to avoid biases or preconceived notions of the culture being observed. For this paper, we defined ‘field notes’ as recorded observations by an individual investigator based on online content they evaluated.

Individual investigators were each assigned to evaluate six months of content from one business’ social media profile, either Facebook or Instagram. For each social media profile, an investigator was immersed in the profile’s content for approximately 3 weeks to collect 6 months of data. Investigators used a template to collect field notes throughout the 3-week immersion period. The template prompted for insights specifically on major themes, tone, and potential target audience for each business’s content. An example of this template is included in Additional file 1. Raw data from the sites was also collected and available for investigators to refer to within field notes and in later synthesis of the notes. A set of field notes for each business profile was created at the end of each immersion period.

#### Stage 2 – preliminary reconstructive analysis

Stage 2 of the Carspecken framework involves analysis and review of the compiled data [43]. This step also involves reflection on the cultural context and site of observation, including relations between participants, or in the case of this study, businesses. During this stage, initial impressions of themes are documented and areas are identified that may need further exploration in proceeding stages.

Two investigators created field notes for each social media business profile, each observing a distinct 6 months of content. Therefore, four different investigators in total completed field notes for each businesses’ content on Facebook and Instagram to produce a final set of compiled field notes covering all content from that business over a one-year period. This approach was meant to bolster observations through triangulation of data, as well as to reduce biases from any individual observers [44]. After data collection, all investigators met for a debriefing meeting to discuss experiences and initial thoughts on larger themes. This step allowed investigators to consider and document big picture ideas and themes before delving into detailed data analysis. A list was then complied of initial themes.

#### Stage 3 – dialogical data generation

Stage 3 involves a dialogical approach to gain an insider’s position with respect to culture [43]. This is unique from Stages 1 and 2, which solely utilize an outsider perspective. This step also involves making connections between data points and checking the data against initial themes identified in Stage 2.

This was a multi-site study involving two social media sites, therefore data were collected for individual cannabis businesses’ Facebook and Instagram profiles. Analysis was focused on the individual business, not on distinct social media platforms, as our goal was to capture a more holistic picture of the businesses’ interaction with social media across multiple sites. Two investigators read through all compiled field notes and constructed lists of themes individually, informed by initial themes identified by the group in Stage 2 and responses to the data. Three investigators met and came to consensus on a combined list of proposed themes based on the individually constructed lists of themes.

#### Stage 4 – describing system relations

During this stage, themes are extracted from the data, providing evidence to support proposed themes from the primary data [43]. A full description of the documented relationships and typical events is developed. Reflexivity is also involved in this stage, where investigators reflect upon their own biases and assumptions to prevent them from influencing the study.

Dedoose (Dedoose, Los Angeles, California), a qualitative analysis software, was used to upload field notes and then extract themes from the text. Dedoose allows the user to create a list of variable and highlights excerpts of uploaded text to apply theme to the text directly. Multiple users can apply themes blinded to other users’ analysis, and these can be unblinded after analysis is complete for comparison. Three investigators were involved in thematic analysis. The investigators utilized a constant comparative approach [44]. Inductive reasoning based in grounded theory guided theme identification [45].

In the first cycle of analysis, two investigators independently applied themes to combined field notes from one business, blinded to the other’s analysis. After applying categories to this transcript, the investigators met and the selected text was reviewed with themes un-blinded. The investigators discussed and reached consensus on theme and subtheme nomenclature for the combined list of themes during this stage, as well as any additions or revisions to the themes list. The categorization process was then applied to combined field notes for a second business. The purpose of this second review was to evaluate reliability and validity of the proposed themes. After review, discussion and achieving consensus on the themes, the themes were applied to all remaining data using this constant comparative approach. Throughout this process, the third investigator was available to help reach consensus if the two primary investigators were not in agreement.

#### Stage 5 – system relations as explained by findings

An important part of ethnographic research and the Carspecken framework is to create a narrative that describes the specific culture that was observed [43]. Our goal for this study was to identify ways cannabis businesses cultivate an online presence and exert influence that may reach youth; therefore, findings were analyzed through the lens of this goal. This analytic step aims to develop a thick description through the process of qualitative analysis. Qualitative analysis data and immersive experience were reflected upon to determine the most important themes. Narratives for major themes were constructed using our experience as observers, qualitative analysis data and process, and all available collected data from investigators. In this way, themes became interconnected to create a cohesive picture.

## Results

A total of 14 businesses were evaluated across both Facebook and Instagram, resulting in 14 sets of combined field notes. Six social media profiles from six businesses were deactivated after the launch of our study, so field notes for these profiles were based on less than a full year of content. Table 1 displays demographic information for each of the cannabis business profiles. The major theme that we identified to represent the data was Normalization of Cannabis, subthemes included strategies for this through 1) Broad Appeal and 2) Specific Targeting.

**Table 1:** Descriptive information for recreational cannabis business profiles on Instagram and Facebook

Companies	Instagram followers	Facebook followers	Location	City Setting Classification
Business A	644	286	Sitka, Alaska	Rural
Business B	3467	5351	Boulder, Colorado	Urban
Business C	4459	11,129	Denver, Colorado	Urban
Business D	5456	2868	Denver, Colorado	Urban
Business E	6741	5747	Denver, Colorado	Urban
Business F	1129	161	Scappoose, Oregon	Rural
Business G	1191	1232	Eugene, Oregon	Urban
Business H	1650	652	Portland, Oregon	Urban
Business I	1755	1484	Bend, Oregon	Urban
Business J	9897	1913	Portland, Oregon	Urban
Business K	850	1923	Mount Vernon, Washington	Rural
Business L	898	421	Spokane, Washington	Urban
Business M	1060	452	Seattle, Washington	Urban
Business N	1333	1664	Aberdeen, Washington	Rural

Displays demographic information for the cannabis businesses in our sample, including number of Instagram followers, number of Facebook followers, city and state location, and city setting classification based on population using U.S. census classification definitions

### Normalization of Cannabis

Normalization of Cannabis emerged as the central theme in our observation of cannabis marketing content on social media. Normalization of Cannabis was observed through two distinct approaches, which we designated as subthemes of Broad Appeal and Specific Targeting. Several smaller categories emerged within those subthemes, explained in more depth in the following paragraphs, with example posts from each category displayed in Table 2.

**Table 2:**
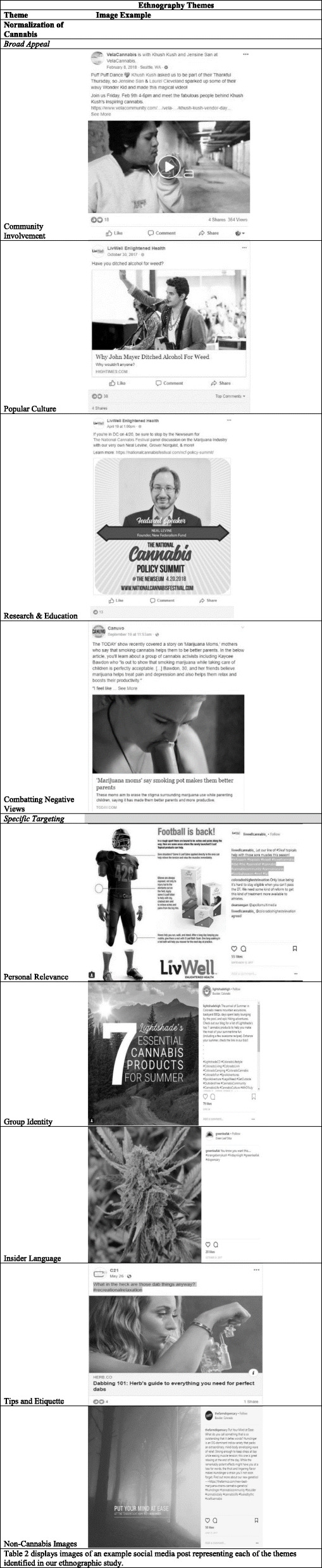
Ethnography Themes

Displays images of an example social media post representing each of the themes identified in our ethnographic study

### Broad appeal

Our first of two subthemes was Broad Appeal, a common marketing strategy used by cannabis businesses as a way to normalize their product and appeal to the largest possible audience. Broad Appeal methods of normalizing cannabis included Community Involvement, connections to Popular Culture, Research and Education, and changing stereotypes through Combatting Negative Views.

#### Community involvement

Community Involvement as a way to normalize cannabis use was defined as acknowledging or thanking customers, supporting local businesses, and volunteering/doing charity work in the community. Examples included posting photos of employees volunteering at a local business or attending local events. Consumers were also encouraged to attend community events and “shop locally”.

#### Popular culture

Popular Culture was defined as cultural activities or products generally recognized by most people and often, aimed at youth [25, 46]. Connections to Popular Culture were illustrated through the use of celebrities such as mainstream musicians that were sometimes connected with the promotion of concerts or other events. Other references to Popular Culture included use of memes, bitmojis, and humor that may appeal to youth. One specific post depicted a chicken crossing the road towards a cannabis business with the caption, “The real reason he crossed the road.”

Connections to Popular Culture were also made by inserting cannabis into non-cannabis context. This approach was defined as altering existing or easily recognizable content to include the use of cannabis, it often incorporated images. One example post featured an image of the Statue of Liberty consuming cannabis; a second example post displayed a meme of a 1950s family sitting at home with a woman asking, “Who wants bong rips and coffee?” Trendy hashtags were also utilized as a way to tap into existing online communities that were unrelated to cannabis. Examples included use of #weedcrushwednesday instead of #womancrushwednesday, a popular social media hashtag used by adolescents to call out women they find attractive as well as use of the hashtag, #girlscoutcookies even when not referencing this specific strain of cannabis.

#### Research and education

Research and Education was defined as posts that exposed the business engaging in practices designed to educate the general public about the benefits of cannabis as well as promote political and scientific discussion among the cannabis community. Examples included sharing news articles promoting medicinal or recreational benefits along with hosting free, educational seminars for the public.

#### Combatting negative views

Another category within the Broad Appeal subtheme was Combating Negative Views. One approach was by making fun of the “stoner stereotype” as examples included, “Today’s cannabis user is not always what you’d expect” and “Spreading ideas about “stoner” normalization.”

### Specific targeting

Our second subtheme was Specific Targeting. Specific Targeting was defined as targeted messages designed to appeal to specific people, populations, or interests. Sub-categories included Personal Relevance, Group Identity, Insider Language, Tips and Etiquette, and Non-Cannabis Images.

#### Personal relevance

On a micro level, Personal Relevance was defined as associating cannabis use with specific demographics and hobbies, perhaps to motivate consumers to view cannabis products as personally relevant. Cannabis use was featured on social media in combination with normal, everyday activities such as running, yoga, cooking, and the outdoors. Examples included sponsoring a “cannathlete” runner as a brand ambassador for the store, promoting events at a local yoga studio, and blogging about ways to incorporate cannabis into weekly recipes. Specific demographics were also targeted, including youth, older adults, females, moms, and pet owners.

#### Group identity

On a macro level, Group Identity helped consumers to feel included, as though they were part of a larger community centered around cannabis. References to a Group Identity appeared in the form of hashtags and use of slang, such as #cannabiscommunity and #bluntculture as well as inclusive language (e.g. #farmily which references the cannabis farming community as a type of family and using pronouns such as “us, we, our”). These messages were reinforced through repetition, demonstrated through constant use of more general hashtags such as #cannabiscommunity and more specific, local hashtags such as #spokanecannabis. Additionally, several cannabis businesses sold merchandise, encouraging consumers to take a photo of themselves wearing the product and tagging the business with the store hashtag.

#### Insider language

This category represented specific targeting of experienced cannabis users. Insider Language around cannabis use appeared in the form of using many detailed and technical terms related to cannabis and use methods. This category also included the use of language to describe cannabis products and methods of consumption that would likely only be familiar to users, such as slang or insider terms. Other examples included talking about cannabis as a reward or gift for family and friends during the holidays, or implying that use was expected or the norm. Additionally, posts using insider language around the reader’s interest in cannabis use were included in this category. An example of this type of post can be seen in Table 2.

#### Tips and etiquette

This category represented specific targeting of likely new users, as well as users trying new methods. Tips and Etiquette around cannabis use included suggestions on how to incorporate cannabis in small ways into specific hobbies or an everyday lifestyle along with social etiquette around smoking. Some posts appealed to a specific target audience while others were less specific to appeal to more potential customers.

#### Non-cannabis imagery

This category represented attempts to target specific areas of interest that were unrelated to cannabis. Several cannabis businesses promoted the use of text and images unrelated to cannabis, such as aesthetically pleasing images of nature and art. Cannabis use was consistently connected with unrelated themes including animals, nature, friends, and happiness, without much reference to specific types of cannabis that would appeal more to current users.

## Discussion

In this study, we used cyber-ethnography to identify a major theme in cannabis marketing content on social media relevant to youth exposure. This theme was Normalization of Cannabis through the subthemes of Broad Appeal and Specific Targeting.

Our first finding was that cannabis businesses focus on the normalization of cannabis use in social media marketing through a variety of messages. Acceptance of cannabis use has been rising in the U.S. adolescent population over the past few years [7, 8]. Legalization of retail, non-medical cannabis may be a contributing factor, as well as medical uses of cannabis, since these may alter perceptions around safety. Content around legalization may come in the form of social media posts, similar to those evaluated in this study. The findings of this study suggest that cannabis business content may influence acceptability of cannabis use. For example, Broad Appeal forms of cannabis normalization identified in this study, such as Research and Education, may influence youth acceptability of cannabis. An article in *Forbes* [47] described how cannabis companies can use strategies to build online communities by using hashtags and visual media towards “redefining the stoner stereotype.” These strategies may represent an attempt at normalizing cannabis by cannabis businesses, including Combatting Negative Views, which was identified as a subcategory in this study. Additionally, some findings of this study indicate that cannabis marketing attempts to connect cannabis use with Popular Culture and youth-focused activities, such as team sports as seen in Table 2. As a previous study has shown that the majority of youth (78.5%) who reside in states with legalized non-medical cannabis encounter cannabis marketing online, this exposure may alter youth perceptions of cannabis use on a large scale [48].

In our study, we identified many posts that indirectly attempted to shift cannabis stereotypes, for example by referencing cannabis businesses volunteering in the community, as well as directly addressing stereotypes by challenging and mocking them or presenting counter-evidence. This may represent a shift in cannabis culture and a unique aspect of the cannabis industry: unification across businesses to promote a central message around promoting legalization. It may be surprising to those that work with youth that cannabis business social media content is focused on acceptable, community-oriented content rather than counter-culture, rebellious content. Thus, it may be important for parents, health care providers, and substance use prevention workers to be aware of the strategies used by cannabis businesses online.

Our second major finding was that cannabis businesses appeal to a variety of audiences, varying from broad to narrow, many of which may appeal to youth. Content describing Tips and Etiquette for cannabis use and Non-Cannabis Imagery may be used to entice new users, while also exposing them to a strong community and culture around cannabis. For example, Tips and Etiquette around using cannabis and different types of consumption methods may act as a gateway for youth who are not very familiar with cannabis use to enter an “in-group” represented by cannabis slang used in posts. Similarly, content using Group Identity approaches to represent an “in-group” may be aimed at current users, but could also draw in youth by normalizing habitual use. This type of in-group marketing may be particularly influential to youth [49]. Additionally, our findings include Popular Culture references that specifically relate to youth, including humor and memes or hashtags that are popular among youth [13]. Youth may be susceptible to a variety of content marketing strategies used by cannabis businesses. This is concerning given youth are a high-risk group for Cannabis Use Disorder and would facelegal consequences given cannabis use is illegal for youth in all states. While this study did not focus on content defined as appealing to youth by state advertising regulations, this content has been identified in a previous study in online cannabis marketing [31]. The goal of this study was to identify content that may appeal to and/or normalize cannabis use for youth, which is currently not considered a violation of state advertising regulations. The findings of our study could be used to expand the definition of youth appeal in state cannabis advertising policy in efforts to protect youth.

Another important contribution of this paper is the description of a novel methodological approach to evaluating content online. This approach may be particularly useful for describing the influence and strategies of content related to substance use that youth may be exposed to in future research. While a previous study used content analysis to assess adherence to advertising regulations by non-medical cannabis businesses in Washington [31], the current study represents the first effort to qualitatively analyze online cannabis marketing content. Our cyber-ethnographic approach resolves many issues with aspects of this content that cannot be captured using content analysis, which requires pre-defined categories with narrow definitions and can make it difficult to capture more complex or emergent themes. Cyber-ethnography helps resolve this issue, as it can be used with text-based content to identify themes after data collection which allows for more complexity across data analysis. For example, categories such as Community Involvement and Combatting Negative Views identified in this study would have been difficult to define a priori for a content analysis study. Further, the theme of Group Identity, which was central to many of our conclusions, included a wide variety of content that would not have been captured in a discrete, pre-determined definition.

Additionally, the specific complete observer approach used in this study may be useful for understanding a specific subset of content online. Focusing on an open-ended form of content analysis allowed for observation of all features of posts and social media profiles rather than discrete interactions with an individual person. The application of the Carspecken framework can be useful for similar studies as the steps align well with assessing social media content. Thus, the complete observer application of cyber-ethnography to social media may be a useful approach for future research analyzing social media content.

This study has a few important limitations to consider. Investigators were immersed in online content for a limited time period of 3 weeks. It may be of interest for future studies to have investigators immersed in online content for a longer time period (6 weeks or more) to obtain a more complete picture of that business’ overall content. This study only captured publicly available content on Facebook and Instagram, and business profiles were identified with a limited range of keywords. It is possible that a larger variety of keywords may have identified additional or alternative business profiles. The complete observer approach used in this study may limit the ability to establish rapport and immerse oneself in the field. This method was chosen as investigators were interested in the ways in which youth may experience cannabis marketing on social media, which may involve primarily viewing the content rather than interacting with the business. Future research could focus on active participation ethnography to create a better understanding of how youth may interact with cannabis businesses through social media such as by liking and sharing posts. Furthermore, as with all ethnography and qualitative approaches, the researcher’s worldview may influence data collection and interpretation. Incorporating more than one researcher for each business may have reduced the potential for an individual’s bias.

There are implications of the themes identified in this study on youth perceptions of cannabis. First, the focus by cannabis businesses on normalization may have a significant impact on youth perceptions of cannabis use for those exposed to these messages. Health practitioners, educators, and prevention specialists who work with youth should be aware of the potential influence of marketing tactics used online to promote cannabis use. Understanding this content could help those in these roles teach youth to be more critical of marketing to avoid harmful influence. Additionally, much of the specific targeting exhibited by cannabis businesses on social media may appeal to youth and their interests, such as in-group marketing and popular culture references. This finding could be used to strengthen cannabis advertising policies and regulation to monitor online content that may specifically apply to youth.

## Conclusions

Youth are active and engaged digital media users; thus, it is important to consider the implications of youth exposure to influential marketing, including cannabis marketing. More unique approaches, such as the one presented here, are needed to assess strategies used by cannabis businesses to promote and normalize cannabis use in ways that may influence youth.

## Supplementary Information

**Additional file 1.** Ethnography Template and Example.

## Data Availability

The datasets generated and analyzed during the current study are not publicly available to protect the anonymity of the businesses included in our sample but are available from the corresponding author on reasonable request.

## Abbreviations

U.S.: United States
D.C.: District of Columbia

## References

[1] Recreational Marijuana Oregon: Oregon Liquor Control Commission, Division 25; 2017 [Available from: https://www.oregon.gov/olcc/marijuana/Documents/Rules/OAR_845_Div_25_RecreationalMarijuana.pdf. Accessed 5 June 2019.

[2] AAC AAC. Regulations for the Marijuana Control Board 2019 [Available from: https://www.commerce.alaska.gov/web/Portals/9/pub/MCB/StatutesAndRegulations/MarijuanaRegulations.pdf. Accessed 5 June 2019.

[3] Retail Marijuana Code 1 CCR 212–2. In: Department of Revenue MED, editor. Colorado. 2013. https://www.colorado.gov/pacific/sites/default/files/ColoradoRegister.pdf1%20CCR%20212%202%20Retail%20Effective%2002022018.pdf.

[4] 314–55-155 Advertising requirements and promotional items- Coupons, giveaways, etc: Washington State Legislature; 2013 [Available from: https://apps.leg.wa.gov/wac/default.aspx?cite=314-55-155&pdf=true. Accessed 5 June 2019.

[5] Anderson M, Jiang J (2018). Teens, social media, and technology 2018.

[6] Williams S. 3 Hidden Issues With Legalizing Marijuana in the U.S.: Nasdaq; 2021 [Available from: https://www.nasdaq.com/articles/3-hidden-issues-with-legalizing-marijuana-in-the-u.s.-2021-01-08. Accessed 5 June 2019.

[7] Johnston LD, O’Malley PM, Bachman JG, Schulenberg JE, Miech RA (2014). Monitoring the Future national survey results on drug use, 1975-2013: Volume I, Secondary school students.

[8] Chadi N, Hadland SE (2018). Adolescents and perceived riskiness of marijuana: why care?. J Adolesc Health.

[9] Buckner JD, Ecker AH, Cohen AS (2010). Mental health problems and interest in marijuana treatment among marijuana-using college students. Addict Behav.

[10] Green KM, Doherty EE, Stuart EA, Ensminger ME (2010). Does heavy adolescent marijuana use lead to criminal involvement in adulthood? Evidence from a multiwave longitudinal study of urban African Americans. Drug and alcohol dependence. 112.

[11] Beverly HK, Castro Y, Opara I (2019). Age of first marijuana use and its Impact on Education attainment and employment status. J Drug Issues.

[12] Hawke LD, Koyama E, Henderson J (2018). Cannabis use, other substance use, and co-occurring mental health concerns among youth presenting for substance use treatment services: sex and age differences. J Subst Abus Treat.

[13] Richter L, Pugh BS, Ball SA (2017). Assessing the risk of marijuana use disorder among adolescents and adults who use marijuana. Am J Drug Alcohol Abuse.

[14] Bourque A. Under the Influence of Instagram: Cannabis in the Age of Social Media: Forbes; 2019 [Available from: https://www.forbes.com/sites/andrebourque/2019/05/06/under-the-influence-of-instagram-cannabis-in-the-age-of-social-media/#6197a90f4746. Accessed 5 June 2019.

[15] Jernigan DH, Ostroff J, Ross C (2005). Alcohol advertising and youth: a measured approach. J Public Health Policy.

[16] Siegel M, Kurland RP, Castrini M, Morse C, de Groot A, Retamozo C, Roberts SP, Ross CS, Jernigan DH (2016). Potential youth exposure to alcohol advertising on the internet: a study of internet versions of popular television programs. J Subst Use.

[17] Dunlop S, Freeman B, Perez D (2016). Exposure to internet-based tobacco advertising and branding: results from population surveys of Australian youth 2010-2013. J Med Internet Res.

[18] Austin EW, Chen MJ, Grube JW (2006). How does alcohol advertising influence underage drinking? The role of desirability, identification and skepticism. J Adolescent Health.

[19] McClure AC, Gabrielli J, Sargent JD, Tanski SE (2018). Aspirational brand choice and underage alcohol use. J Stud Alcohol Drugs.

[20] Arnett JJ, Terhanian G (1998). Adolescents' responses to cigarette advertisements: links between exposure, liking, and the appeal of smoking. Tob Control.

[21] Turco RM (1997). Effects of exposure to cigarette advertisements on Adolescents' attitudes toward Smoking1. J Appl Soc Psychol.

[22] Brainard B (2003). Companies Exploiting Unregulated Internet to Sell Alcohol, Tobacco Products, Study Finds.

[23] Hong T, Cody MJ (2002). Presence of pro-tobacco messages on the web. J Health Commun.

[24] Nicholls J (2012). Everyday, everywhere: alcohol marketing and social media--current trends. Alcohol Alcohol.

[25] Pediatrics AAo (2010). Children, adolescents, substance abuse, and the media. J Am Acad Pediatrics.

[26] Whitehill JM, Trangenstein PJ, Jenkins MC, Jernigan DH, Moreno MA. Exposure to cannabis marketing in social and traditional media and past-year use among adolescents in states with legal retail cannabis. J Adolescent Health. 2020;66(2):247-54. 10.1016/j.jadohealth.2019.08.024 PMC6980270 31708374

[27] Krauss MJ, Sowles SJ, Sehi A, Spitznagel EL, Berg CJ, Bierut LJ (2017). Marijuana advertising exposure among current marijuana users in the U.S. Drug Alcohol Dependence.

[28] Moreno MA, Gower AD, Jenkins MC, Kerr B, Gritton J (2018). Marijuana promotions on social media: adolescents' views on prevention strategies. Subst Abuse Treat Prev Policy.

[29] Cavazos-Rehg PA, Krauss MJ, Sowles SJ, Murphy GM, Bierut LJ (2018). Exposure to and content of marijuana product reviews. Prev Sci.

[30] Lamy FR, Daniulaityte R, Sheth A, Nahhas RW, Martins SS, Boyer EW (2016). "Those edibles hit hard": Exploration of Twitter data on cannabis edibles in the U.S. Drug Alcohol Dependence.

[31] Moreno MA, Gower AD, Jenkins MC, Scheck J, Sohal J, Kerr B, Young HN, Cox E (2018). Social media posts by recreational marijuana companies and administrative code regulations in Washington state. JAMA Netw Open.

[32] Brewer JD (2000). Ethnography.

[33] Murthy D (2008). Digital ethnography: an examination of the use of new technologies for social research. Sociology..

[34] Hine CM (2000). Virtual ethnography.

[35] Rybas N, Gajjala R (2007). Developing cyberethnographic research methods for understanding digitally mediated identities. Forum Qualitative Sozialforschung/Forum: Qualitative Social Research.

[36] Kowalski M, Hooker C, Barratt MJ (2019). Should we smoke it for you as well? An ethnographic analysis of a drug cryptomarket environment. Int J Drug Policy.

[37] Creswell JW, Poth CN (2016). Qualitative inquiry and research design: choosing among five approaches fourth ed.

[38] Nakamura K (2013). Making sense of sensory ethnography: the sensual and the multisensory. Am Anthropol.

[39] Hardon A, Hymans TD (2014). Ethnographies of youth drug use in Asia. Int J Drug Policy.

[40] Griffiths R, Casswell S (2010). Intoxigenic digital spaces? Youth, social networking sites and alcohol marketing. Drug Alcohol Rev.

[41] Lenhart A. Teens, Social Media & Technology Overview 2015 http://www.pewinternet.org/2015/04/09/teens-social-media-technology-2015/: Pew Research Center; 2015. Accessed 5 June 2019.

[42] Kawulich BB (2005). Participant observation as a data collection mMod. Forum Qualitative Sozialforschung/Forum: Qualitative Social Research.

[43] Carspecken FP (2013). Critical ethnography in educational research: a theoretical and practical guide.

[44] Jonsen K, Jehn KA (2009). Using triangulation to validate themes in qualitative studies. Qual Res Organ Manage Int J.

[45] Glaser BG, Strauss AL (2017). The discovery of grounded theory: strategies for qualitative research.

[46] Valkenburg PM, Piotrowski JT (2017). Plugged in: How media attract and affect youth.

[47] Weed J. Best Practices: Cannabis Executives Share Social Media Advice: Forbes; 2017 [Available from: https://www.forbes.com/sites/julieweed/2017/07/16/best-practices-cannabis-executives-share-social-media-advice/#43e563eb2e72. Accessed 5 June 2019.

[48] Whitehill JM, Trangenstein PJ, Jenkins MC, Jernigan DH, Moreno MA. Exposure to cannabis marketing in social and traditional media and past-year use among adolescents in states with legal retail cannabis.. J Adolescent Health. 2020;66(2):247–54. 10.1016/j.jadohealth.2019.08.024. 10.1016/j.jadohealth.2019.08.024 PMC6980270 31708374

[49] McClure AC, Stoolmiller M, Tanski SE, Engels RC, Sargent JD (2013). Alcohol marketing receptivity, marketing-specific cognitions, and underage binge drinking. Alcohol Clin Exp Res.

