# Supplementary material for: Cyber-ethnography of cannabis marketing on social media
Source: Subst Abuse Treat Prev Policy. 2021 Apr 26;16:35. doi: 10.1186/s13011-021-00359-w (PMC8074195; doi:10.1186/s13011-021-00359-w)
Supplement: Supplementary file 1 — Additional file 1. Ethnography Template and Example. [file 13011_2021_359_MOESM1_ESM.docx]

**Appendix 1: Ethnography Template and Example**

This appendix includes the prompts given to researchers participating in the ethnography study, including example responses.

1. **Which page (business and platform) were you assigned to?**

Business X on Facebook

1. **What main *themes* did you notice from this business’s posts? (~100 words)**

Business X was celebrating the month of September as their “Birthday Month” with several posts reminding consumers of exclusive birthday promotions. The page would also use pronouns like “us, we or our” to explain the store which created a sense of community within the store and its employees. Refers to cannabis as “delicious” making it seem more appealing to first time users or regular customers and “treat yo self,” treat your family and friends- promoting marijuana use as a reward.

1. **What do you feel are the main *values* of this business after coding their posts? (~100 words)**

The store seems to value their customers by offering promotional discounts and deals. Posts are colorful and flashy which will catch the attentions of new users. They also want to seem welcoming towards new users by inviting viewers to their store “come celebrate our birthday with us”. They also want to make the store as accessible as possible with directions/locations of the store linked in many posts, references to store hours, persistent updates on store restocks for previous/current customers, and encouraging consumers to visit the store.

1. **Please describe a *typical customer* that you think this business is targeting or trying to appeal to with these advertisements. (~100 words)**

Adults/Young Adults (early twenties) that are already customers to the store as well as new users. Birthday month seems to target new users due to month long discounts, as well as previous users due to direct references to restocked products. Responsible users due to disclaimer in every post and models over the legal age of use in photos.

1. **Anything else you think is pertinent about this business’s posts? (tone, trends, etc.)**

Associating MJ use with an active outdoor lifestyle. Using youthful female models. Associating specific days of the week with deals- Wednesday 10% off paraphernalia. Linking MJ consumption with sports- basketball and local events- Hoopfest weekend; catering to consumers who enjoy sports/athletes/live an active lifestyle.
